# A Complicated Case of Rheumatoid Arthritis in Septic Shock

**DOI:** 10.7759/cureus.25712

**Published:** 2022-06-07

**Authors:** Annalee Mora

**Affiliations:** 1 Primary Care/Urgent Care, Medical Center Health System ProCare, Odessa, USA

**Keywords:** immuno suppresion, port catheter, peri-prosthetic joint infection, rheumatoid arthritis, organ failure from sepsis

## Abstract

Sepsis is significantly associated with increased mortality among hospitalized patients. Patients can deteriorate rapidly, leading to septic shock (i.e., tissue hypoperfusion and organ dysfunction despite fluid resuscitation that can ultimately require a vasopressor). Patients immunocompromised from medical conditions such as rheumatoid arthritis with multiple joint arthroplasties are at a major risk of increased infections. Equally, medications that impair the immune system's normal function make this clinical case challenging. As noted in this case of a patient with a complex medical history and nontypical sepsis presentation, early intervention and a multidisciplinary approach to patient care is vital to patient improvement and survival during septic shock.

## Introduction

According to the World Health Organization, sepsis causes one in five deaths worldwide [1]. It is one of the main healthcare issues leading to high mortality and disability. Life-threatening organ dysfunction is caused by a dysregulated host response to infection [2]. An upregulation of pro- and anti-inflammatory pathways leads to a widespread release of cytokines, mediators, and pathogen-related molecules that activate coagulation and complement cascades [3]. The unregulated inflammatory immune response is most frequently due to a severe complication of a localized infection that can become systemic if not recognized early and managed promptly. Sepsis is becoming more prevalent among the aging population, immunocompromised patients, patients with frequently performed arthroplasties, and with the increasing use of implantable devices, emergence of life-sustaining technologies, and increased resistance to antimicrobial medications [4,5].

Sepsis can progress into septic shock, leading to profound circulatory, cellular, and metabolic abnormalities associated with a greater risk of hospital mortality of up to 60% [2,3,6]. These persistent systemic inflammatory responses at the capillary endothelial level cause vasodilation, capillary permeability, thrombosis, tissue hypoperfusion, and, eventually, organ failure. These can elevate lactate levels and cause metabolic acidosis and severe hypotension, requiring vasopressor therapy despite fluid resuscitation. Therefore, early identification of infection and aggressive goal-directed therapy are crucial in preventing deterioration that can improve outcomes.

## Case presentation

A 61-year-old Hispanic woman was transferred from a regional county hospital to our facility for an acute level of care with septic shock and non-ST-elevation myocardial infarction. A day prior, she presented to the regional county emergency department (ED) due to generalized weakness and inability to walk, with lower back and right hip pain. She received a steroid injection, to which she had usually responded well for past similar symptoms. However, her body weakness worsened, and she was generally not feeling well. She went back to the ED the following day, where she was found to be hypotensive, tachycardic, and hypoxic on room air but was afebrile. She had no headache, chest pain, palpitations, shortness of breath, rhinorrhea, abdominal pain, dysuria, hematuria, nausea, or vomiting. Her white blood cell (WBC) count was significantly high with an elevated troponin level. She received two liters of intravenous (IV) crystalloids, ceftriaxone IV, and aspirin, and she was started on a heparin drip and subsequently IV dopamine. An attempt to wean off dopamine was unsuccessful. Due to the continual drop in her blood pressure, dopamine was continued en route to the transferring facility.

She had a significant medical history of rheumatoid arthritis (RA) and polyarthritis. She took daily prednisone and was on a tocilizumab infusion every four weeks via a port-a-catheter. After bilateral hip and bilateral knee arthroplasties, she developed chronic bilateral hip pain and was on hydrocodone as needed. Socially, her chronic medical conditions and the recent death of her grandson had brought her into feelings of depression, for which she was taking escitalopram. She was diagnosed with Hodgkin's lymphoma six years ago, but was in remission. She is also on ondansetron as needed for nausea or vomiting.

On arrival, her physical examination revealed a temperature of 36.7°C and a heart rate (HR) of 78 beats/min (bpm), with a sinus rhythm. Her blood pressure was 143/76 mmHg, and her respiration rate was 17 breaths/min, with unlabored breathing. Her oxygen saturation level on oxygen at 3 L was 98%. She was awake, alert, oriented, and in no acute distress. Her head was normocephalic with no evidence of trauma. The lungs were clear to auscultation bilaterally without tenderness, and a port-a-catheter was present on the left anterior chest. No heart murmurs were detected. The patient had active bowel sounds. We noted moderate to severe arthritic changes with deformities in both hands and feet. Pedal edema was not present upon inspection.

The initial workup in the ED was remarkable for 33,000 leucocytes with increased neutrophils, low hemoglobin, low platelets, low sodium, high potassium, high blood urea nitrogen (BUN), high creatinine, high blood glucose, low carbon dioxide, and elevated liver enzymes (alkaline phosphatase, aspartate transaminase, alanine transaminase), albumin, C-reactive protein, procalcitonin, and troponin (Table 1). The urine analysis was cloudy with WBCs and a trace amount of leukocytes. Her arterial blood gas showed metabolic acidosis (i.e., low pH and a low bicarbonate level). Additionally, her chest X-ray showed bilateral interstitial changes. Her electrocardiogram and lactic acid levels were unremarkable.

**Table 1:** Patient's laboratory investigations WBC, white blood cell; Hgb, hemoglobin; Hct, hematocrit; CO_2_, carbon dioxide; BUN, blood urea nitrogen; AST, aspartate transaminase; ALT, alanine transaminase; ALP, alkaline phosphatase; CRP, C-reactive protein; ABG, arterial blood gas; HCO_3_, bicarbonate; HPF, high-power field

Analyte	Initial values	Day 3	Day 10	Day 15	Reference range
WBC (x 10^3^/µL)	33	10.7	28.2	5.4	4.0-12.0
Hgb (g/dL)	11.1	9.4	5.9	8	12.0-15.0
Hct (%)	33.8	29.9	19	23.5	36.0-45.0
Platelet count (x 10^3^/µL)	125	91.4	240	223	150-400
Band Neutrophils (%)	36	2	18		0-4
Sodium (mmol/L)	133	143	141	143	136-145
Potassium (mmol/L)	5.2	4.6	4.3	4	3.5-5.1
CO_2_ (mg/dL)	13	23	27	32	21-32
BUN (mg/dL)	53	28	14	15	7-18
Creatinine (mg/dL)	2	1	0.16	0.7	0.60-1.30
Glucose (mg/dL)	252	170	245	85	70-110
Hemoglobin A1c (%)	5.6				<5.7%
Total bilirubin (mg/dL)	2.8	0.4	0.3	0.4	0.2-1.0
AST (U/L)	101	20	38	10	15-37
ALT (U/L)	165	88	46	22	12-78
ALP (U/L)	215	129	95	29	50-136
Troponin (pg/dL)	1254		23		3.0-58.9
CRP (mg/dL)	2.26				0.29-0.90
Procalcitonin (ng/ml)	1.25		0.24		0.00-0.50
Lactic acid (mmol/L)	1.9				0.5-2.2
Urine color	Cloudy				Yellow
Leukocyte esterase	Trace				Negative
Urine WBC (/HPF)	10				0.5
Urine ketones (mg/dL)	Trace				Negative
ABG pH	7.173				7.350-7.450
HCO_3 _(mEq/L)	12.3				22-28

A central line was inserted upon admission, and the patient was started on IV fluids. We discontinued dopamine and started norepinephrine. The patient was in septic shock. Due to the high probability of bacteremia, we initiated broad-spectrum antibiotics (vancomycin, piperacillin/tazobactam, and levofloxacin). A one-time dose of sodium bicarbonate was also administered. The patient was admitted to the intensive care unit in sepsis protocol.

**Figure 1:**
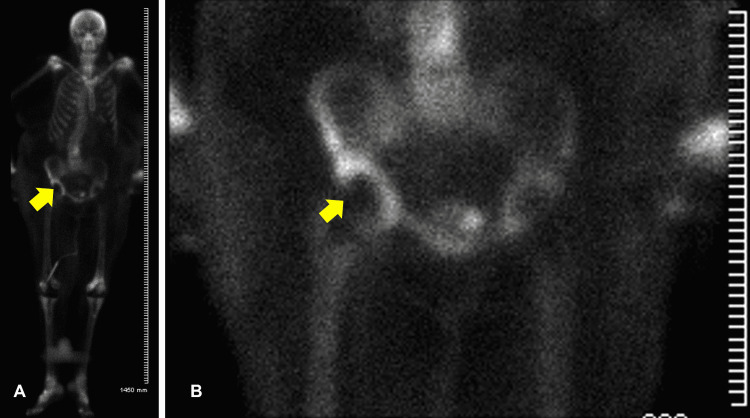
Whole body (A) and right hip (B) scan

Other diagnostic tests showed echogenicity visualized on the port-a-catheter line in transthoracic echocardiogram (TTE), but we later confirmed no vegetation on the transesophageal echocardiogram (TEE). Her right hip X-ray showed right total hip arthroplasty with a small nondisplaced fracture of the acetabular wall, later confirmed by computed tomography of the hips showing what appeared to be chronic insufficiency fractures in the right acetabulum with moderate hip joint effusion decompressing into the greater trochanteric bursa and chronic tears of gluteal tendons. An increased uptake over the right hip was seen in the bone scan (Figure 1). The orthopedic surgeon was consulted. Right hip aspiration was subsequently done to determine the source of infection but did not show any growth. A urine culture resulted in no growth as well. Peripheral blood cultures were positive for *Staphylococcus aureus*, in which case antibiotics were narrowed based on sensitivity to vancomycin and cefepime. The other test results were unremarkable.

**Figure 2:**
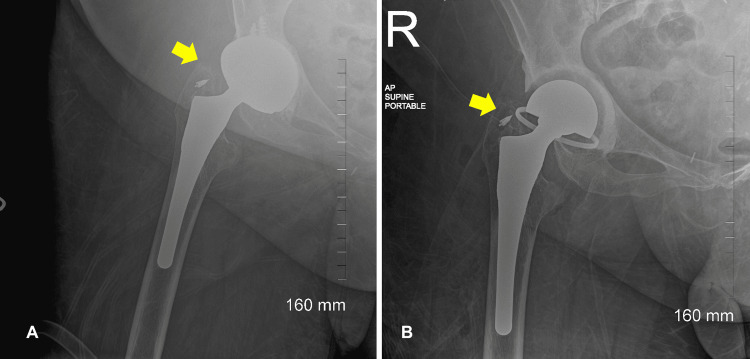
Right hip X-ray before (A) and after (B) debridement and revision

The patient's general condition improved after three days. The WBC count and liver and kidney enzyme levels were much better (Table 1). On hospital day 6, the patient was transferred to the medical floor in a stable condition, and IV antibiotics were continued. On day 9, the patient started reporting concerns of severe pain over the right hip and thigh with associated swelling and warmth but without fever. An urgent venous duplex scan showed no evidence of deep vein thrombosis. A repeat blood count showed an increased WBC count, while hemoglobin and hematocrit levels were decreased. Two units of packed red blood cells were transfused. The orthopedic surgeon was consulted a second time. The patient was taken to the operating room for irrigation, debridement, and partial revision of the right hip for a prosthetic joint abscess. Figure 2 presents the X-ray of the right hip before and after debridement and revision. Approximately 250 ccs of frank pus was drained from the hip. Cultured samples positively confirmed methicillin-sensitive *S. aureus*, comparable with her initial blood sample results, and antibiotics were narrowed to vancomycin.

Postoperatively, her condition improved, and she continued vancomycin. The WBC normalized two days postoperatively. Her hemoglobin and hematocrit improved. Repeat blood cultures showed no growth. She was monitored regularly via follow-up by the orthopedic surgeon in the medical unit. She was started on physical therapy and tolerated it well. Her overall condition improved, and a discharge plan was made. A social worker was involved in her care. The patient decided to be transferred to a skilled nursing facility in her hometown to be closer to her family. The patient was advised to follow up with her primary care physician, rheumatologist, and infectious disease specialist.

## Discussion

This case describes a patient in sepsis followed by a continuum of septic shock. In general, there are multiple sources of sepsis. Although respiratory, genitourinary, gastrointestinal, and skin or soft tissue infections account for 80% of sources of infection, implantable venous access ports, and total joint arthroplasties are also likely potential sources of life-threatening sepsis [5,7,8]. In this case, patient's permanent port-a-catheter was implanted 10 years ago for long-term access to immunosuppressive therapy (tocilizumab). The permanent venous port has low extravasation rates and infection, ranging from 0.6% to 27% [4]. However, patients with immunocompromised states have a greater risk for the rate of infections leading to colonization at the insertion site and bloodstream infection. Staphylococcus is the most common causative microorganism [4].

Additionally, the patient was on a chronic corticosteroid, increasing the risk of infections due to long-term immunosuppression. The echogenicity visualized on the port-a-catheter line during TTE was suspicious for a vegetation. TTE was performed initially because it was a noninvasive test. With the high suspicion of infective endocarditis, TEE was subsequently done for better sensitivity and better image quality, but the case was ultimately confirmed negative for vegetation.

Moreover, patients with underlying inflammatory joint diseases are at a high risk of developing septic arthritis. Prosthetic joint infections can increase the infection twofold [9,10]. Total joint arthroplasty is one of the most reliable and common orthopedic procedures that can improve the quality of life in patients with degenerative joint diseases. However, prosthetic joint infection is a significant complication. The bacteria can adhere to the prosthetic surfaces that form biofilms, leading to increased resistance to the immune system and antimicrobials. Staphylococcus is frequently implicated as the causative microorganism of septic arthritis and prosthetic joint infections [11-13].

Septic arthritis is typically monoarticular and presents with warmth, swelling, and painful joints with limitation of activity [10]. These symptoms were all manifested by the patient. The patient's existing comorbidities of RA, joint arthroplasties, corticosteroid use, and biologic disease-modifying antirheumatic drug use increased her risk of septic arthritis. Ongoing urinary tract infections also increase the risk of prosthetic joint infections through contiguous spread, which was likely in this patient [13]. Aspirated synovial fluid is valuable for the early diagnosis of prosthetic joint infections. However, it has a sensitivity of 72% [13]. The result of the patient's aspirated culture was negative. Intraoperative synovial fluid culture can identify pathogens in about 20% of culture-negative patients [14]. This was eventually required in the patient because the synovial fluid culture result was negative. For optimal management of prosthetic joint infections, both surgical intervention and antimicrobial therapy are usually required [13]. Therefore, debridement, irrigation, and partial revision of the right hip were surgically performed. The goal of the procedure was to remove all infected or necrotic soft tissue, restore a pain-free function of the infected joint, and promote rapid recovery [10]. Given the patient's underlying comorbidities, long-term antimicrobial therapy may be warranted.

Based on history, the patient had multiple underlying risk factors for developing sepsis and septic shock. She developed multiorgan involvement, as manifested by acute kidney injury (elevated BUN, creatinine, and potassium), liver dysfunction (elevated liver enzymes), cardiac dysfunction (elevated troponin level), acute hypoxic respiratory failure, anion gap metabolic acidosis, and persistent hypotension despite fluid resuscitation, and required vasopressor therapy.

In 2016, the international guidelines updated the definition of sepsis and septic shock with the Sepsis-3 criteria for greater consistency and to facilitate early recognition and timely management [2]. The Sequential Organ Failure Assessment (SOFA) is a useful scoring system for adult patients with sepsis that assesses the performance of several organ systems in the body; the higher the score, the more likely a patient to have a poorer outcome of sepsis. The quick SOFA (qSOFA) uses three elements (i.e., systolic blood pressure ≤100 mmHg, respiratory rate ≥22 breaths per minute, or altered mentation) and can rapidly be used at the bedside.

A patient with sepsis typically presents with a temperature of >38°C or <36°C, HR >90 bpm, respiration rate >20 breaths per minute, and WBC count >12,000/mm^3^ or <4000/mm^3^. However, the presentation of sepsis can be variable and often depends on the source of infection [5]. This patient did not present with fever, possibly due to the anti-inflammatory effect of the prednisone that she took daily. Steroid use influences the biosynthesis and release of various agents involved in inflammation [15]. Her serum lactate level was 1.9 mmol/L, below the diagnostic criterion for septic shock in Sepsis-3 [7]. This could be because the patient received 2 L of IV fluids several hours before transfer. Evidently, in the early hours (the first six hours), her lactate levels normalized rapidly following the initiation of therapy [16].

The patient's elevated troponin level was likely secondary to demand ischemia because of hypoperfusion and hypoxia. The procalcitonin level was also elevated. Procalcitonin is an essential biomarker of infection and its severity, especially for bacterial infections [17]. The patient presented initially with mild anemia, likely due to her chronic medical conditions. However, it gradually worsened during her hospital stay and postoperatively, which required blood transfusions. Anemia is one of the most common complications in patients with sepsis [18]. It is caused by hemodilution, iatrogenic blood loss, decreases in iron supply, erythropoietin production, and erythrocyte lifespan that is usually mild to moderately severe [18].

## Conclusions

Early recognition of sepsis is vital to improving patient outcomes and decreasing mortality. An infection is the most common cause of the dysregulated bodily response that can deteriorate rapidly into a multiorgan failure. A goal-directed therapy with the first signs of sepsis should be initiated using protocols and sepsis care bundles. This can be achieved by providing hemodynamic support through IV fluids and vasopressor therapy. Similarly, empiric antimicrobials should not be delayed. As seen in this case, a patient who presents with complicated medical conditions can be challenging. The interdisciplinary team approach in providing decisions and support through diverse skills and perspectives in providing the best possible care is vital for patients' overall improvement and survival in sepsis and septic shock.
